# A qualitative study of people who use methamphetamine during the early COVID-19 pandemic to inform future ED harm reduction strategies

**DOI:** 10.1186/s12245-023-00505-0

**Published:** 2023-04-28

**Authors:** Sophie C. Morse, Callan Fockele, Ly Ngoc Huynh, Alina Zatzick, Lauren Kirsten Whiteside

**Affiliations:** Department of Emergency Medicine, University of Washington, Seattle, WA USA

**Keywords:** Methamphetamine, COVID-19, Emergency, Harm reduction

## Abstract

**Background:**

Morbidity and mortality rates related to methamphetamine are on the rise. Simultaneously, social-distancing guidelines were issued in March 2020 to decrease transmission of COVID-19. The aim of this study was to explore concerns regarding methamphetamine use during the COVID-19 pandemic and subsequent harm reduction strategies with patients who use methamphetamine to inform emergency department (ED)-based harm reduction approaches.

**Methods:**

A mixed-methods study of adults residing in Washington with high-risk methamphetamine use and a recent ED visit from April-September 2020 was performed. Participants completed a survey and a semi-structured interview on perceptions and experiences of COVID-19. Descriptive statistics were used for survey responses. Interview transcripts were analyzed and guided by modified grounded theory using an iterative approach to refine the guide and codebook. Interviews were independently coded by 2 investigators and discussed until consensus.

**Results:**

Twenty-five participants completed the survey; 20 participants were interviewed (45% recently used heroin, 40% unstably housed). Thirty-five percent was worried about COVID-19 infection. Three themes emerged from the interviews: (1) increase in meth use, (2) interplay of meth obtention and COVID-19, and (3) interactions with healthcare and social services.

**Conclusions:**

People who use methamphetamine noted an increase in use along with the social distancing guidelines put in place for COVID-19 and employed a variety of harm reduction profiles when obtaining methamphetamine. Also, the pandemic brought difficulties in accessing care and amplified mistrust in healthcare instructions and public health messages. Based on these qualitative interviews, further work should consider aligning methamphetamine and COVID-19 harm reduction messages and working with trusted community resources to improve harm reduction strategies for methamphetamine use and COVID-19. IRB: Informed Consent by the University of Washington Human Subjects Division (approval number, STUDY00009277).

**Supplementary Information:**

The online version contains supplementary material available at 10.1186/s12245-023-00505-0.

## Background

Prior to COVID-19, methamphetamine use was on the rise with increasing rates of overdose [1, 2], treatment admission [3], and emergency department (ED) visits [4]. Between April 2019 and October 2020, the Drug Abuse Warning Network noted that 33.7% of substance-use-related ED visits were due to methamphetamine use [5]. Although acute methamphetamine intoxication or overdose has not been dose related [6] as it has with other substances like opioids, it has been related with cardiovascular complications, ranging from palpitations to NSTEMIs, as well as hyperthermia, subarachnoid hemorrhage, kidney problems, rhabdomyolysis, and consequences of psychiatric symptoms such as agitation or paranoia that may lead to life-endangering behaviors [7–10]. Overdose related to methamphetamine is on the rise, and fatal overdoses due to psychostimulants [2] have increased [11, 12].

Using drugs alone is the main risk for both fatal and nonfatal overdose, especially when used with other substances [1, 2]. However, this overdose risk reduction guideline is in direct contrast with social distancing guidelines limiting social gatherings and encouraging shelter in place [13–15] to help decrease transmission from COVID-19. Additionally, there are several risk factors for continuing use including the following: increasing dose, taking higher risks to obtain substances, or resuming substance use after a period of abstinence which includes loneliness, self-isolation, and financial or economic stress [14, 16–18]. These risk factors can exacerbate psychiatric disorders which can sometimes lead to substance use, as a way to cope [14, 16–18]. The SARS-COV-2 coronavirus (COVID-19) pandemic exacerbated these trends as people who use methamphetamine demonstrate many risk factors associated with developing a poor outcome due to COVID-19 infection [19–21]. Chronic conditions such as lung disease, cardiac disease, and HIV are common in people who use methamphetamine and are associated with additional healthcare needs [22, 23], severe illness from COVID-19 [24–26], or mistaking withdrawal symptoms as COVID-19, due to inability to access substances [14, 27].

A widening socioeconomic vulnerability due to the pandemic may exacerbate healthcare inequities for people who use methamphetamine [20]. These vulnerabilities may include the inability to isolate due to unstable housing, loss of social support, and a decrease in social services [14, 21, 25, 28]. Moreover, while attempting to minimize the spread of COVID-19, the social distance guidelines put forth by 42 states and territories [29] further restricted access to outpatient healthcare, forcing many to avoid or delay healthcare for fear of getting infected with COVID-19 during an appointment [14, 30, 31] or present to the ED [14, 32–34]. The ED can be a location where people can access low-barrier healthcare and, therefore, is a location to transmit public health messages [35]. More so, the ED may be an important healthcare location for providing public health messages given continued in-person healthcare when other locations have closed or adjusted their service delivery model [14, 32, 33, 36–39].

Given the rise in methamphetamine use at a time that the COVID-19 pandemic continues, it is crucial to understand how people who use methamphetamine view their personal risk for contracting COVID-19, as well as how use has changed during the pandemic [14, 32, 33, 36–39]. Therefore, the objective of this study was to describe the impact of COVID-19 on people who use methamphetamine and to determine how substance use has changed among this group, to inform ED-based harm reduction messages that can reduce methamphetamine-related harms, as well as reduce risk from COVID-19 for this vulnerable population.

## Methods

This study aimed to determine COVID-19 risk mitigation strategies among people who currently use methamphetamine and to consider ways to tailor public health messaging from the ED for this population using semi-structured interviews.

Surveys and interviews were completed in Washington, from April 2020 to September 2020, beginning within a month of the first case of COVID-19 and the signing of Washington’s governor’s “stay-at-home” order in March 2020 [40]. The study was approved with a waiver of written documentation of informed consent by the University of Washington Human Subjects Division (approval number: STUDY00009277), and a Certificate of Confidentiality was obtained from the National Institute of Health (NIH).

Possible participants were screened and recruited through convenience and respondent-driven sampling; 47 participants having responded to the community flyers completed the screening survey. Of those, three participants were seeds, referring three participants, two of which completed the interview, while the third completed only the survey. The recommenders were not compensated for their referrals; those referred received the same compensation as other participants. Flyers were sent to local syringe-exchange services, substance use treatment clinics, peer support groups, supportive housing, facilities, and outpatient-based opioid treatment clinics. Possible participants contacted study staff by phone and were verbally screened for eligibility to answer questions about motivations and experiences while using methamphetamine as well as questions about COVID-19 and associated behaviors [41]. Eligible participants were adults residing in Washington state, with access to a phone, who had an ED visit for any reason in the last 3 months, who used methamphetamine in the last 30 days, and met criteria for moderate or high-risk methamphetamine use disorder by having a NIDA-modified-ASSIST score of four or higher [42]. Possible participants who called the phone but did not meet these criteria were not eligible to complete the survey or interview. Eligible participants then provided verbal informed consent and completed a survey with a trained research assistant who directly entered data into a REDCap [43] database. Participants received a US $5 gift card for completing the survey. After the first 10 participants were interviewed, we continued interviews with eligible participants selecting for people with diverse demographics. For the semi-structured interview, participants provided verbal consent to be audio recorded. Next, we performed semi-structured interviews with invited participants to elucidate opinions related to COVID-19 and associated risk mitigation strategies. The interview guide is provided as Appendix A.

Participants who completed the semi-structured interview received a US $25 gift card.

A single-item question was used to assess past 30-day methamphetamine use for eligibility by asking the following: “In the past 30 days, how often have you used meth?” Next, participants were also asked by the NIDA-modified ASSIST [42] to determine the risk for methamphetamine use disorder. Information was obtained on age, sex, gender, education, employment, and housing. Patients were asked single-item questions about tobacco, vaping, and alcohol. Comorbid conditions, such as Generalized Anxiety Disorder and Major Depressive Disorder, were assessed using validated scales. The PHQ-2 [44] was used to determine the past two-week depression, and the GAD-2 [45] was used to determine the past two-week generalized anxiety disorder. Participants were asked about perception of likelihood, severity, and worry for developing COVID-19 with questions adapted from prior surveys on other infectious diseases [46]. Participants were also asked to identify their preferred source of health information.

Descriptive statistics were used to report participant characteristics and survey responses. Qualitative data were collected and analyzed simultaneously using a modified grounded theory approach [47]. All interviews proceeded over the phone with the study research coordinator (L. H.), who was trained in qualitative interviewing and trauma-informed care. There was no familiarity between the interviewer and interviewees prior to this study. The interview guide was refined during the three initial interviews before being finalized. After the three initial interviews, the study research coordinator (L. H.) and two research assistants (S. M. and A. Z.) experienced in substance use, and emergency medicine research developed a codebook from this data. The codebook was iteratively refined until consensus was achieved among this group. The remaining 17 transcripts were independently coded by S. M. and L. H. with the principal investigator (L. W.) as an arbitrator, of which 11 were also independently coded by A. Z. All interviews were recorded, transcribed, and uploaded to Dedoose [48] for analysis. A grounded theory approach guided the initial process, and a constant comparative analysis process was used to code the interviews [47]. The final sample of 20 participants was determined when the interviewer (L. H.) and research team (S. M. and L. W.) noted data adequacy and saturation of themes.

## Results

Forty-seven people completed the eligibility survey. Of those, 31 were eligible, 25 completed the survey, and 21 completed the interview. Twenty interviews were transcribed and coded. One interview was not transcribed and not included due to poor sound quality and a difficult connection that could not be resolved; thus, 20 participants were included in the analysis. Overall, interviewed participants had a mean age of 42 years (standard deviation, 9 years); a majority of the participants were male (*n* = 14, 70%); 30% (*n* = 6) were Black; 10% (*n* = 2) were American Indian/Alaska Native; and 40% (*n* = 8) were currently homeless. Almost all (*n* = 19, 95%) of the participants had health insurance, and the majority (*n* = 18, 90%) of participants reported current anxiety and/or depression. Similarly, of those interviewed, many used other drugs with 70% (*n* = 14) using heroin at least once in their lifetime and 45% (*n* = 9) reporting current use in the past 3 months (Table 1).

**Table 1:** Demographics, clinical, and substance use characteristics (*n* = 20)

Variable	Interviewed *n* = 20 (%)
**Age (mean ± SD)**	41.5 ± 9
**Age (median, IQR)**	42.5, 11
**Male sex**	14 (70)
**Female sex**	6 (30)
**Gender “other”**	3 (15)
**Race**^**a**^	
White	12 (60)
Black	6 (30)
American Indian/Alaska Native	2 (10)
Hispanic/Latino	4 (20)
Preferred not to answer	1 (5)
**Other demographics (yes)**	
Unemployed	13 (65)
Currently homeless	8 (40)
Living in a home or apartment alone	8 (40)
Living in a home or apartment with others	4 (20)
**Highest level of education**	
Completed more than high school	9 (43)
**Has medical insurance (yes)**	19 (95)
**Comorbidities and co-use**	
Anxiety and/or depression in the past 2 weeks	18 (90)
**Current substance use (30** **days)**	
Smoke cigarettes or vape e-cigarettes (yes)	15 (75)
Drink alcohol (yes)	10 (50)
Heroin (yes)	9 (45)
Injected in the last month (yes)	13 (65)

^a^Participants can select more than one answer

In the sample population, 60% (*n* = 12) participants were concerned about COVID-19’s symptom severity on their health. However, 35% (*n* = 7) did not perceive a high likelihood of getting infected with COVID-19, and 35% (*n* = 7) reported being “somewhat” or “extremely” worried about getting infected. Nearly two-thirds (*n* = 13, 65%) of participants reported the Internet as the place to get information about COVID-19, whereas nobody reported their personal doctor as the main source of information. In contrast, 35% got health-related issues from the Internet, and 25% sought this information from a personal doctor (Table 2).

**Table 2:** COVID-19 health attitudes

Variable	Interviewed *n* = 20 (%)
**COVID-19**	
If you were to get infected with COVID-19 in the next 12 months, how serious of a health issue would that be for you? (Moderately and extremely concerned)	12 (60)
How likely are you to get infected with COVID-19 in the next 12 months? (Somewhat and very likely)	7 (35)
How worried are you about getting infected with COVID-19 in the next 12 months? (Somewhat and extremely worried)	7 (35)
**Vaccine acceptance**	
Did you receive an influenza vaccine last year (Fall 2018-Spring 2019)?	9 (45)
Did you receive or plan to receive an influenza vaccine this year (after August 2019)?	5 (25)
If a COVID-19 vaccine becomes available for public use this year, would you get vaccinated? (Yes)	10 (67)^a^
**Where do you get most of your information on health-related issues?**	
Family/friends	2 (10)
Government agencies	4 (20)
Internet	7 (35)
Personal doctor	5 (25)
Television/newspaper	2 (10)
Other	0 (0)
**Where do you get most of your information on the COVID-19 virus?**	
Family/friends	2 (10)
Government agencies	4 (20)
Internet	13 (65)
Personal doctor	0 (0)
Television/newspaper	1 (5)
Other	0 (0)

^a^*n* = 15. As for the first five people who were interviewed, there was no vaccine available

Three major themes of (1) increase in use, (2) interplay of meth obtention and COVID-19, and (3) interactions with health care and other social services emerged from the qualitative analysis.

### Theme 1: increase in use

When stay-at-home orders were implemented in Washington state, participants reported increased methamphetamine use to cope with the isolation, manage boredom, and tolerate the changes or loss of routine. Many interviewees reported increasing methamphetamine use to tolerate isolation brought upon by social distancing guidelines. Many participants identified this increased consumption to cope as a manner of self-medicating to adhere to the changing guidelines*.*

Meth is a drug that causes you to socially isolate and social distancing, … and people are paranoid and so I don't think this is … that's why it's been sort of easy to go back into using is because it helps me to follow these rules a lot better. (Male, 38, White, living in a home or apartment alone).

Next, participants reported using meth to deal with the loss of activities, which lead to feelings of boredom. Many stated that increasing their use of meth helped occupy time.

Boredom is not good for me, being bored. Stuck inside. […] I ain't got nothing to do … I ain't got nothing to do tomorrow. (Male, 50, African American or Black, living in home or apartment with others).

Lastly, many participants reported a disruption in the routines, social networks, recovery support groups, and hobbies that aided in recovery and were associated with reducing use. One participant described their routine prior to COVID-19 and noted the closure of community spaces where they engaged with others led to a return to use.

I used to go to this place on [street], I might go sit in a group or something just to be doing something. Just go over there, visit with my case manager and stuff like that. […] I ain't smoking no meth.… I ain't trying to be all high off meth. But [now] there ain't nothing there to do. And then when all this shit start[ed], like they closed the building on [street], they closed […] other shit, that's when I started smoking more. (Male, 50, African American or Black, living in home or apartment with others).

Another participant noted that the closure of businesses has led to the loss of his job and the loss of routine which led to a resumption of methamphetamine use.

Just when the Governor ordered the ‘stay at home, stay healthy’ order, I no longer had a job, was getting paid, had no responsibilities. And isolation and social distancing have never been healthy things for me, so staying at home is not really staying healthy for me; it's usually a quick line to bad habits. And so within a couple weeks of staying at home and staying healthy, I started using again. (Male, 38, White, living in home or apartment alone).

### Theme 2: interplay of methamphetamine use and COVID-19

For many participants, patterns of methamphetamine use and other activities related to substance use continued as usual, with no changes pertaining to purchasing methamphetamines or interactions with dealers, as many participants were not worried or concerned about COVID-19 infection.

I’m being honest, not really. It hasn't stopped me. It hasn't changed nothing for me (Male, 52, African American or Black, Homeless).

Others noted changes in behaviors around methamphetamine use consistent with public health guidelines and messages to reduce COVID-19 transmission such as decreasing social exposure, limiting sexual partners, or mask wearing. However, even those mitigating COVID-19 risk still described ambivalence around infection by not completely modifying their pattern and manner in obtaining meth.

So I mean I'm still going to take my precautionary measures and wear my mask and use some hand sanitizer and wipe the steering wheel down …I'm still safe as much as I can be about [COVID-19], but I don't worry about it. I'm not honestly thinking about it. (Male, 22, Hispanic or Latino, living in home or apartment with others).

Some were taking COVID-19 risk reduction measures around their methamphetamine use due to worry and concern about getting the virus from community, or social, exposures.

[COVID-19] hasn't changed the amount, but before, if someone wanted to use my pipe and they were putting something in it, I'd be like, okay, now I'm getting some extra stuff in there…but now COVID-19's going around, no thank you. So I don't let people use my pipes. And I try to keep distance. (Female, 50, African American or Black, living in home or apartment alone).

### Theme 3: interactions with healthcare and other social services

The social isolation guidelines implemented to decrease the number of infections lead to the decreased accessibility of appointments in healthcare, as well as in social services. Additionally, the lockdown evoked different responses from the participants in the study.

#### The guidelines are “triggering” due to past experiences

Social distancing restrictions evoked feelings of helplessness and lack of control for some participants, especially those with histories of trauma. Many described being introduced to methamphetamine for the first time in a social context where they had less power. One participant noted that restrictions brought back feelings of being forced to do things they did not want to do. Participants were isolated and left to think about and cope with current and past experiences without social support.

I mean I think there can certainly be PTSD triggers for people during this. Like I know that this is a reminder of a lot of bad past events for me. […] the idea of being forced to do something doesn't really always jive well with me. I know everybody's got their own past and so I imagine that […] a lot is happening here, whether it's big or small, that's triggering different things for different people. (Male, 38, White, living in home or apartment alone).

#### Reduction or delay in access to healthcare services

The arrival of the pandemic greatly restricted in-person healthcare services. Interviewees reported decreased or delayed access to healthcare services, and some expressed worry about the risk of COVID-19 transmission. Several participants rely on public transportation and noted concern of catching the virus on the way to healthcare facilities.

When I get back on the methadone program, I could see the methadone program being like a place that [COVID-19] be easily contractable…. and [on] the public transit (Female, 40, White, Homeless).

Social determinants of health, including housing, were a large source of concern and worry and tightly woven into discussions around wellness. Many patients living in congregate facilities were at higher risk for COVID-19 and recognized this risk.

I’ve been tested four times since March. I am worried about it because I do not live with my husband, but he’s in the shelter right now […] I’m scared he might get it and not know it and the next I’ve got it and don’t know it. (Female, 50, African American or Black, Living in home or apartment alone).

#### Institutional mistrust

Some participants voiced mistrust of information from public health and government officials due to ongoing and historical acts of deceit and oppression from such institutions. This mistrust manifested as suspicions about COVID-19 in general and the impact on health and doubt related to messages from public health institutions about harm reduction strategies.

I think some of the people that I might engage on a daily basis are more at-risk [for COVID-19] and probably could be at a higher risk than the average, but other than that, no, because I wear gloves and mask,[…] but I actually think that this is similar to the Tuskegee experiment…I believe it's a population control. Or it's something to establish fear. (Male, 48, African American or Black, Homeless).

In addition, interviewees noted skepticism about their personal risk of infection and felt their substance use and lifestyle were somehow protective against getting COVID-19. This personal suspicion aligned with overall mistrust of institutions.

I think it's more like that we are bad hosts because we're polluting ourselves and that it [COVID-19] doesn't want to attach, or something … I don't know. (Male, 45, White, Living in home or apartment alone).

#### The inaccessibility of e-recovery

The transition of in-person recovery groups to online platforms was remarkably unfavorable to interviewees. For some participants, the inability to access phones, the Internet, and technology were personal barriers that made attendance in support groups near impossible. Even those who were able to access recovery groups in virtual formats acknowledged the limitations of these recovery resources.

I mean I think that we've seen quite a few … quite a bit of relapsing. The challenge, even though we have Zoom meetings and we still try to maintain a sense of normalcy, the reality is this is not normal. A lot of people in our community don't have access to technology. There may be stigma attached. (Male, 38, White, Living in home or apartment alone).

I am part of some support groups, but Zoom meetings in recovery groups are terrible. [Laughter] So it's been … this has been … it's been a tough time in general just because you can't do the recovery the way you want to do it. And […] it's difficult to find that community right now. (Male, 38, White, Living in home or apartment alone).

#### Lost opportunities to get connected to recovery

COVID-19, and the associated policies to limit social interactions, paused many in-person peer-support groups that support recovery or transitioned these groups to virtual formats. Many participants mentioned difficulty accessing groups or not finding them as fulfilling. The low-barrier in-person nature of drop-in groups was described as critical to meeting people “where they are” in their recovery. Access issues were heightened for those who may benefit the most from support, such as unstably housed individuals or those pre-contemplative about reducing their use.

The group that I lead is usually on [time, day], and I would say oftentimes in that group where we meet in person there's at least one person with less than one week of sobriety, possibly still high, and a good chance of being unhoused. I haven't seen anyone like that [since COVID-19 started]. (Male, 38, White, Living in home or apartment alone).

## Discussion

Overall, concerns about COVID-19 were present in this sample of participants who use methamphetamine and had a recent ED visit. Guidelines and policies put in place in 2020 to reduce the transmission of COVID-19 significantly reduced in-person interactions and closed businesses and public buildings, which changed the environment for everyone. Study participants reported using more methamphetamine but finding fewer opportunities to connect to services, including recovery after the stay-at-home guidelines were issued. Participants also described mistrust in public health and skepticism about COVID-19 and health information. Additionally, the inaccessibility of virtual platforms and telemedicine to initiate or sustain recovery were well described.

Increased substance use to cope with the stress and environmental changes related to COVID-19 is consistent with known trends, and substance use patterns are seen after other disasters. In prior work, people with alcohol use disorder (AUD) were more likely to cope with disaster-related emotional stress by drinking alcohol compared to those without AUD, including those in recovery [49]. However, unlike other naturally occurring (e.g., hurricanes, earthquakes) or human-caused (e.g., mass shooting) disasters, the COVID-19 pandemic has stretched over a year since the President declared it a Nationwide Emergency on March 13, 2020 [50]. Likewise, in our participant sample, the elimination of routine, employment, and changes in housing as well as the application of COVID-19 guidelines may have overlapped with an increase in the use of methamphetamine in this population. People who use drugs such as methamphetamine may experience sequelae directly related to the waxing and waning COVID-19 pandemic for much longer compared to other naturally occurring disasters, such as a hurricane, which have a more concrete acute phase. The increase in methamphetamine use in this study population mirrored an increase in substance-related emergency department (ED) visits during a time when ED volumes were overall down [32, 33, 36–38]. People with substance use disorders have an increased risk of overdose due to the increased chance of using alone [21, 51]. Therefore, EDs and other social service providers need to be flexible and should consider talking to patients about relapse and overdose. Additionally, resources, tailored to substance use and COVID-19 harm reduction, are needed and should be frequently updated to correspond with updated COVID-19 information.

Importantly, the intersectionality of methamphetamine with the resultant precautions and social distancing guidelines to reduce the risk of obtaining COVID-19 was a critical theme. Some participants reported increasing or resuming methamphetamine use to cope with state guidelines, [21, 52] as well as implementing COVID-19 harm reduction strategies such as handwashing, wearing a mask, and limiting in-person contacts [21, 53]. While other healthcare and social service locations closed doors and decreased in-person contacts, the ED remained open and continued to offer in-person healthcare [32, 33, 38, 54]. Harm reduction messages for people who use methamphetamine should overlap with COVID-19 harm reduction messages. The majority of our sample did not get information about COVID-19 or other health issues from their doctor even once social distancing measures were modified. Healthcare providers and other people who provide these messages should be ready to acknowledge that social distancing guidelines to mitigate the risk of COVID-19 are at direct odds with harm reduction messages related to overdose including methamphetamine overdose. Our data suggests limiting in-person interactions [2] to reduce the risk of contracting COVID-19 can also impact recovery.

Next, the inability to access resources was an important theme given the transition from in-person recovery to telehealth options. The state and local guidelines around social distancing and limiting in-person interactions to decrease the COVID-19 transition came with a healthcare transition to telehealth [38]. This was especially true in addiction care, where historically the model of care relied on in-person interactions to obtain health care and medications for addiction. In-person interactions were also paramount for counseling and group meetings which, for many, are central to recovery [2, 55]. Innovations including telehealth allowed for improved access to prescribing buprenorphine for opioid use disorder which helped improve access to treatment [56]. Previous work has documented the benefits of telehealth. Established members in a 12-step narcotics anonymous (NA) group successfully transitioned to online virtual meetings during the pandemic, and the majority of those surveyed found the online meetings effective [57]. However, our work documented a possible gap for people trying to initiate attendance at a group meeting such as NA. It is possible that telehealth is a viable option for established participants but is not a good option for those trying to initiate attendance at a group meeting or for those that lack access to technology [27, 58]. With increasing vaccination rates, community groups should consider offering both forums to allow for patient choice.

### Limitations

While this adds to the literature on exploring concerns of people who use methamphetamine during the COVID-19 pandemic, there are some important limitations to note. First, the sampling frame was used to sample a wide range of perspectives, but due to sample size, results may not generalize to different populations. While we achieved data adequacy and saturation of themes, we acknowledge this small sample size does not allow for generalizability. During the study design, RDS recruitment strategy was attempted; however, only two seeds were successfully enrolled; 90% (*n* = 18) of the interviewed participants were a result of convenience sampling, based on people seeing the flyer in the community and calling in for the screening survey. The target population was people in Washington, which may not represent the experience in other geographic areas. There was a large number of people who use heroin; therefore, some results may be related to drug use that is not representative of methamphetamine use alone. Additionally, participants were recruited and interviewed by phone, thus the study did not capture the experience and opinions of those who did not have access to one. Participants self-reported recent ED visit, which was not confirmed by a review of an electronic health record.

## Conclusion

Participants noted an increase in use with implementation of social distancing guidelines, acknowledgement of risk-mitigation strategies when obtaining methamphetamine, and the struggles to access healthcare. These issues were amplified due to the lack of in-person social support and the transition to e-recovery sources. Moving forward, there is a need for further research and program development. Specific areas that could be targeted include exploring a relationships between healthcare workers, healthcare intuitions, and trusted community champions or liaisons to increase trust in public health messages and the healthcare system. Future work should also determine the best ways to implement these relationships and operationalize harm reduction strategies for COVID-19 and methamphetamine.

## Supplementary Information

**Additional file 1: Appendix 1.** Interview Guide.

## Data Availability

The data generated and analyzed during the study are not publicly available due to the nature of qualitative research and possibility of identifiable information but are available from the corresponding author on reasonable request.

## Abbreviations

ED: Emergency department
Meth: Methamphetamine
COVID-19: SARS-CoV-2 virus
AUD: Alcohol use disorder
HIV: Human immunodeficiency virus
PTSD: Post-traumatic stress disorder
NIDA: National Institute of Drug Abuse
NA: Narcotics anonymous
