# Supplementary material for: A qualitative study of people who use methamphetamine during the early COVID-19 pandemic to inform future ED harm reduction strategies
Source: Int J Emerg Med. 2023 Apr 28;16:30. doi: 10.1186/s12245-023-00505-0 (PMC10139825; doi:10.1186/s12245-023-00505-0)
Supplement: Supplementary file 1 — Additional file 1: Appendix 1. Interview Guide. [file 12245_2023_505_MOESM1_ESM.docx]

**Appendix 1: Interview Guide**

1. COVID has changed life in a way that no one saw coming. How has COVID impacted you?
2. Have you been worried about contracting COVID and have you been tested or tried to get testing for COVID?
3. Has anyone you known gotten COVID?
4. What do you see as your risk factors for COVID?
5. How has COVID changed the way you use drugs?
6. In the last couple of months, how you used more or less of anything?
7. Where do you get drugs from and has that changed?
